# Supplementary material for: Experimental identification of aminomethanol (NH2CH2OH)—the key intermediate in the Strecker Synthesis
Source: Nat Commun. 2022 Jan 19;13:375. doi: 10.1038/s41467-022-27963-z (PMC8770675; doi:10.1038/s41467-022-27963-z)
Supplement: Supplementary file 1 — Supplementary Information [file 41467_2022_27963_MOESM1_ESM.pdf]

Supplementary Information for

Experimental Identification of Aminomethanol (NH_2CH_2OH) – The Key Intermediate in the Strecker Synthesis

Santosh K. Singh[1], Cheng Zhu[1], Jesse La Jeunesse[1], Ryan C. Fortenberry[2*], Ralf I. Kaiser[1*]

[1]*Department of Chemistry, University of Hawaii, 2545 McCarthy Mall, Honolulu, HI 96822, USA.*
W. M. Keck Research Laboratory in Astrochemistry, University of Hawaii, 2545 McCarthy Mall, Honolulu, HI 96822, USA.

[2]*Department of Chemistry and Biochemistry, University of Mississippi, 322 Coulter Hall, University, MS 38677-1848, USA.*

Correspondence to: ralfk@hawaii.edu, r410@olemiss.edu

This PDF file includes:

Supplementary Methods
Supplementary Figures 1 to 5
Supplementary Tables 1 to 5
Supplementary References

Supplementary Methods

1. Calculations of column densities of methylamine (CH_3NH_2) and oxygen (O_2) in the ice mixture.

The column density of methylamine ($N_{CH_3NH_2}$) in the ice mixture was determined to be 5.3 ± 0.5 × 10^{16} molecules cm^{-2} using the integrated area of the IR absorption band ν_3 (2794 cm^{-1}) of methylamine and its corresponding integral absorption coefficient (A_{exp}) of 1.0 × 10^{-18} cm $molecule^{-1}$ in equation 1;[1]

$$N_{CH_3NH_2} = \frac{\ln 10 \int_{\nu 1}^{\nu 2} A_\nu d\nu}{A_{exp}} \frac{\cos(\theta)}{2} \qquad (1)$$

where $\int_{\nu 1}^{\nu 2} A_\nu d\nu$ is the integrated peak area of the absorbance in the region ν_1-ν_2 cm^{-1}, A_{exp} is the integrated absorption coefficient and θ is the angle of incidence (45°) of the IR beam. On the other hand, the column density of the oxygen molecule cannot be directly determined from the FTIR spectrum because its vibrational mode is IR inactive. To determine the column density of O_2, we first calculated the thickness of methylamine (CH_3NH_2) in the ice mixture (CH_3NH_2 + O_2) using equation 2

$$d_{CH_3NH_2} = \frac{\ln 10\, M \int_{\nu 1}^{\nu 2} A_\nu d\nu}{2N_A \rho n\, A_{exp}} \sqrt{n^2 - sin^2\theta} \qquad (2)$$

where M is the molecular weight, N_A is the Avogadro's number, ρ is the density of methylamine ice (0.85 g cm^{-3})[1] and n is the refractive index of methylamine (n = 1.40)[1]. From equation 2, the thickness of methylamine ice in the ice mixture was determined to be 39 ± 4 nm. The total thickness of the ice mixture (d_t = 239 ± 24 nm) was determined using equation 3.

$$d_t = \frac{m\lambda}{2\sqrt{n^2 - \sin^2\varphi}} \qquad (3)$$

Where, m is the number of interference fringes (m= 1) observed after reflection of the He-Ne laser light (λ = 632.8 nm) from the silver substrate and ice surface, φ (= 4°) is the incidence angle of the laser, and n is the average refractive index. Next, we determined the O_2 ice thickness (d_{O_2}) to be 200 ± 20 nm, by subtracting the methylamine ice thickness ($d_{CH_3NH_2}$) from the total thickness (d_t) of the ice mixture (equation 4).

$$d_{O_2} = d_t - d_{CH_3NH_2} \qquad (4)$$

$$N_{O_2} = \frac{d_{O_2} 2N_A \rho n}{M\sqrt{n^2 - sin^2\theta}} \frac{\cos(45°)}{2} \qquad (5)$$

Using thickness ($d_{O_2} = 200\ nm$), density (ρ = 1.54 g cm^{-1})[2] and refractive index (n = 1.25)[2] of O_2 ice in eq. 5, the column density of the oxygen in the ice mixture is determined to be 49 ± 5 × 10^{16} molecules cm^{-2}.

2. Generation of VUV light

(I) 9.50 eV – The second harmonics (532 nm) of a pulsed ND:YAG laser (Spectra Physics, PRO-250-30; 30 Hz, 10 ns) was used to pump a dye laser (Sirah Cobra Stretch) having Rhodamine 610/640 dye mixture. The fundamental output of the dye laser (606.948 nm) undergoes frequency tripling to generate 202.316 nm (ω1) light. Two photons of ω1 is required to access the resonant transition of Krypton. The third harmonics (355 nm) of a second ND:YAG laser (Spectra Physics, PRO-250-30; 30 Hz, 10 ns) was used to pump another dye laser (Sirah Cobra Stretch) containing Coumarin 450 dye to generate 449.794 nm (ω2) light. The 202 nm and 449.794 nm lights were spatially and temporally overlapped on pulsed jet of Krypton (80 µs, 30 Hz) which act as a non-linear medium. Difference frequency mixing of two photons of ω1 and one photon of ω2 in Krypton (2ω1-ω2) results in the generation of 130.51 nm (ω_{VUV} = 9.50 eV) light. A LiF biconvex lens is used to separate the 130.51 nm light from residual 202 nm and 449.794 nm lights.

(II) 9.10 eV – The 136.24 nm (9.10 eV) light was generated via difference frequency mixing of two photons of 222.566 nm (ω1) and one photon of 579.014 nm (ω2) light in xenon. To produce ω1, the fundamental output of a dye laser (445.132, Coumarin 450), pumped by the third harmonics (355 nm) of an ND:YAG laser (Spectra Physics, PRO-250-30; 30 Hz, 10 ns), was frequency doubled through a BBO crystal. The second harmonics (532 nm) of a pulsed ND:YAG laser was used to pump another dye laser having Pyrromethane 593 dye to generate ω2 (579.014 nm). The ω1 and ω2 lights were spatially and temporally overlapped on the pulsed jet of Krypton for difference frequency generation of 136.24 nm light. The generated VUV light (136.24 nm) is eventually separated from the residual 222.566 nm and 579.014 nm lights by the help of a LiF biconvex lens and directed at about 2 mm above the sample to ionize the subliming molecules.

Supplementary Figures

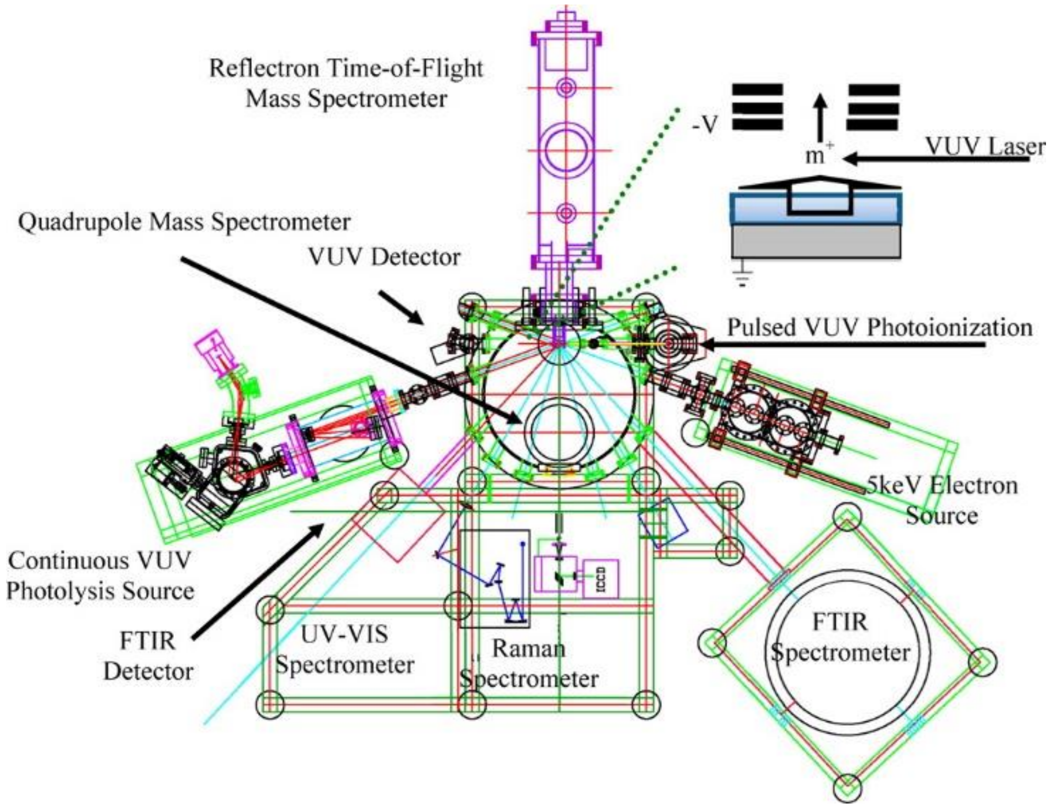

Supplementary Figure 1. Schematic top view of the ultra-high vacuum chamber including the electron source, analytical instruments (FTIR, UV-VIS, ReTOF), and cryogenic target (point of convergence lines).[3,4] Adapted with permission from ref. 3. Copyright (2013) American Chemical Society.

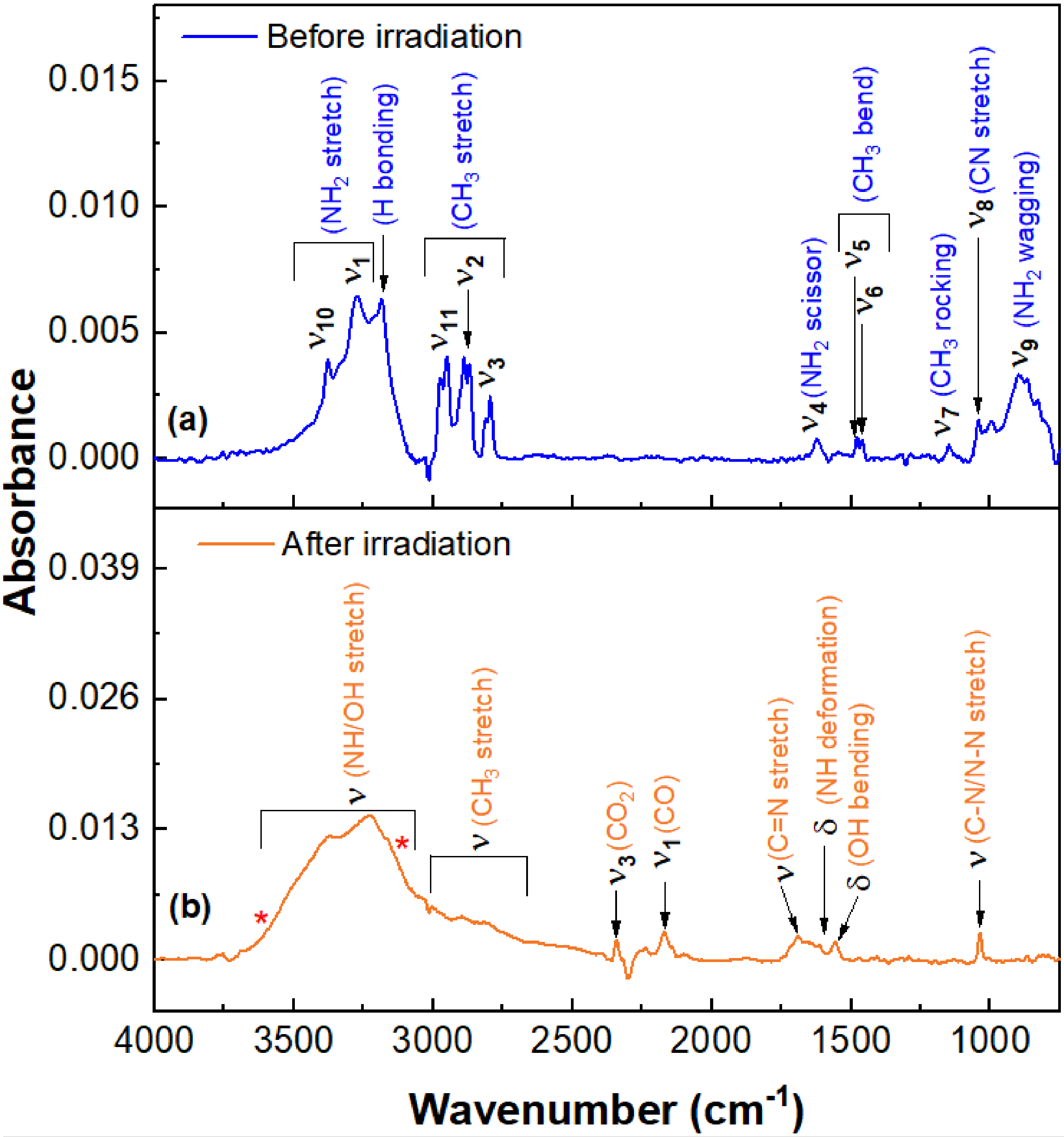

Supplementary Figure 2. Infrared spectra of methylamine and oxygen ice mixture (a) before and (b) after irradiation at 5 K. Vibrational modes of methylamine ($\nu_1 - \nu_{11}$) are labeled in (a). New absorption bands observed after irradiation are labeled in (b). The calculated and scaled -OH (3659 cm^{-1}) and -NH_2 (3146 cm^{-1}) stretching vibrations of aminomethanol (**1**) are marked with asterisks. Scaling factors of 0.96 and 0.95 were used to scale the calculated -OH and -NH_2 stretches of **1**, respectively. These scaling factors were determined by dividing experimental -OH (3656 cm^{-1}) and -NH_2 (3312 cm $^{-1}$) vibrational frequencies of N-methylhydroxylamine[5] (**4**) by corresponding calculated frequencies. The unscaled vibrational frequencies of aminomethanol isomers are provided in Supplementary Data 2. Source data are provided as a Source Data file.

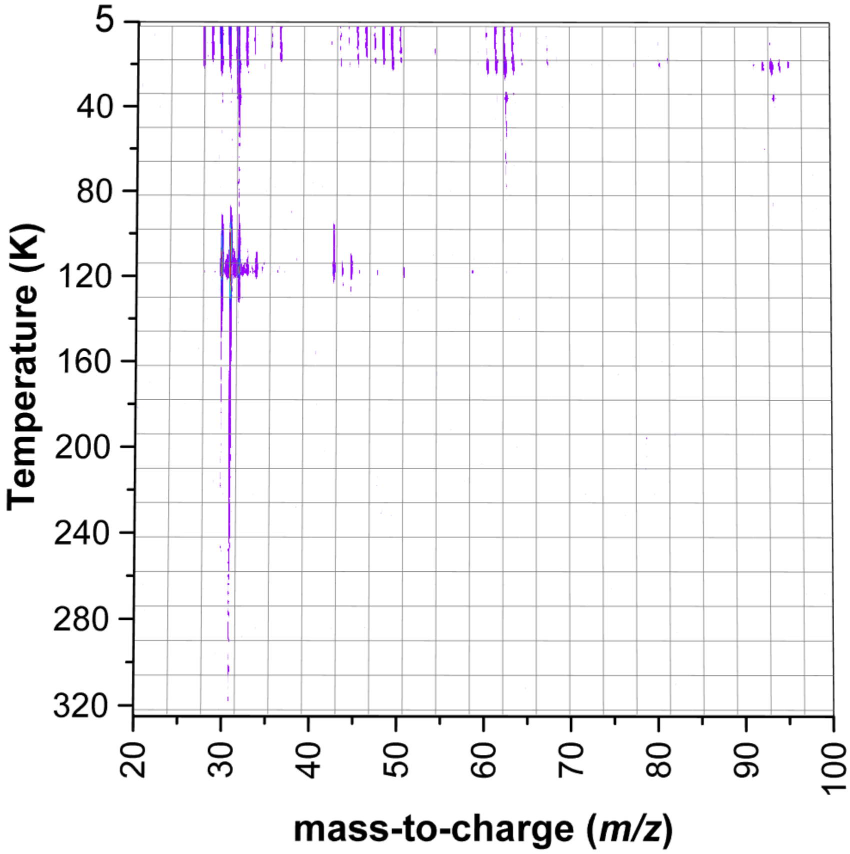

Supplementary Figure 3. PI-ReTOF mass spectrum measured as a function of temperature during the TPD phase of non-irradiated methylamine (CH_3NH_2) and oxygen (O_2) ice mixture at a photon energy of 10.49 eV. The dark colored purple lines indicate ion counts. Source data are provided as a Source Data file.

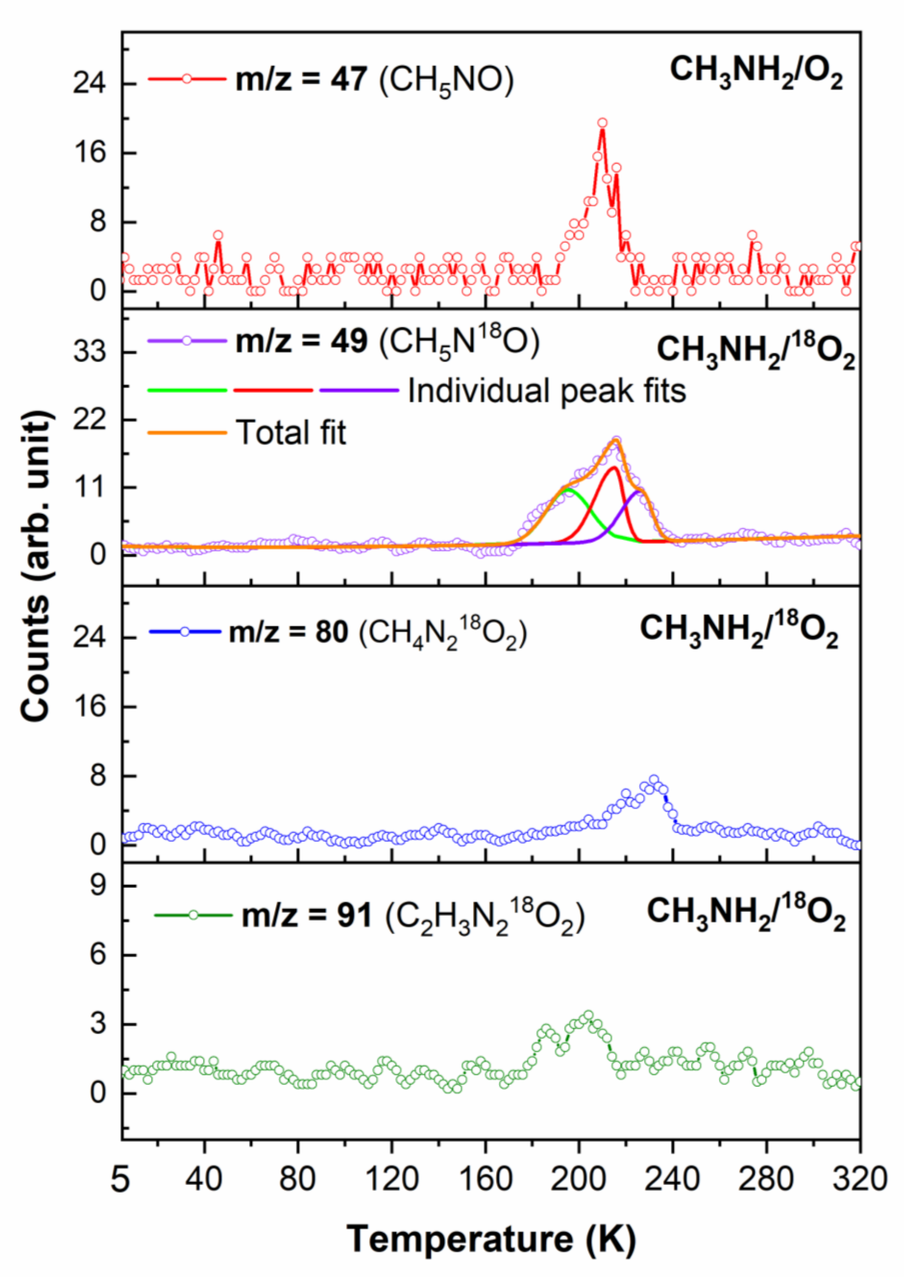

Supplementary Figure 4. PI-ReTOF mass spectra measured at m/z = 49, 80 and 91 during the sublimation phase of irradiated CH_3NH_2 – $^{18}O_2$ ice mixture along with the mass spectrum recorded at m/z = 47 in non-isotopic system (CH_3NH_2 – O_2) at a photon energy of 9.50 eV. Source data are provided as a Source Data file.

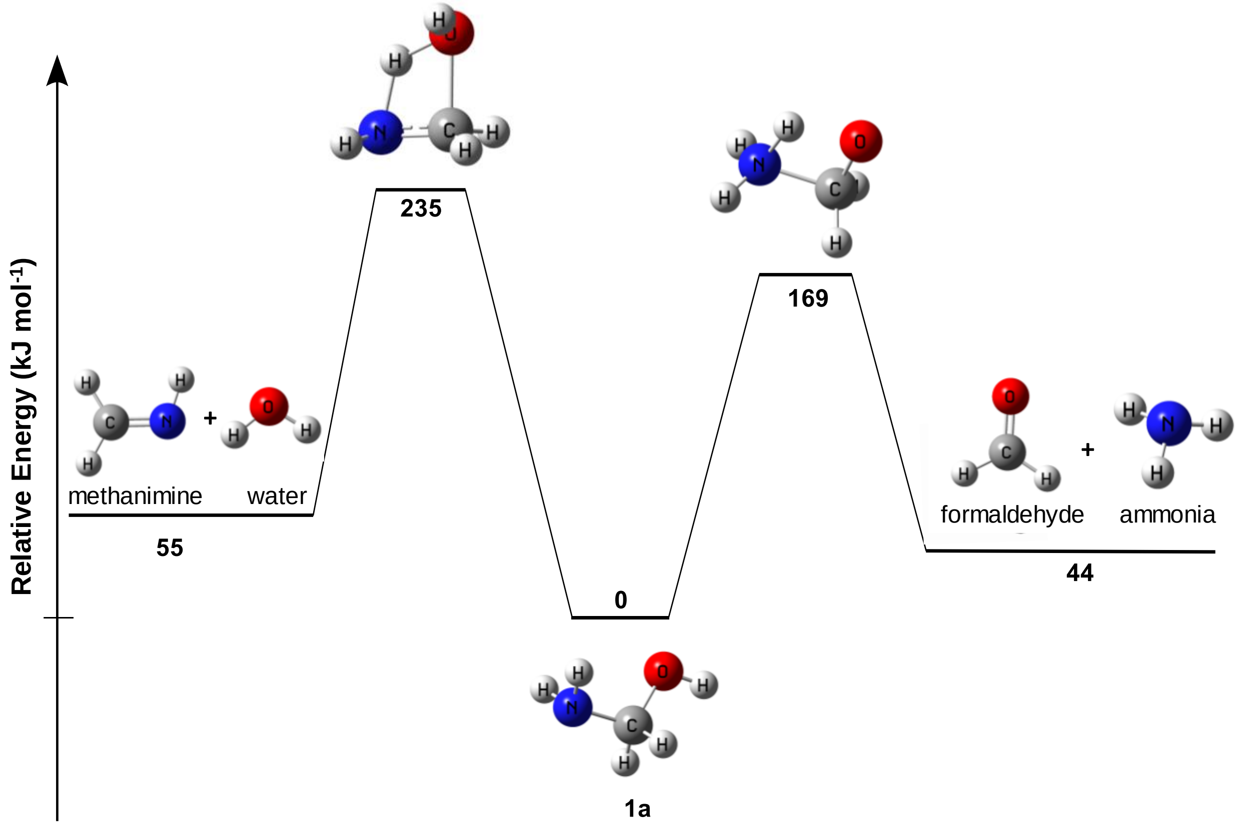

Supplementary Figure 5. Decomposition pathways of aminomethanol calculated at the CCSD(T)/CBS//MP2/aug-cc-pVTZ level of theory. Coordinates of the transition states are provided in Supplementary Table 5.

Supplementary Tables

Supplementary Table 1. Data applied to calculate the average dose per molecule.

Initial Kinetic energy of the electrons	5 keV
Irradiation current (I)	100 ± 10 nA
Irradiation time (t)	3600 s
Average penetration depth, l	202 ± 20 nm
Average kinetic energy of backscattered electrons, $E_{bs}{}^{a}$	3.3 ± 0.3 keV
Fraction of backscattered electrons, $f_{bs}{}^{a}$	0.35 ± 0.04
Average kinetic energy of transmitted electrons, $E_{trans}{}^{a}$,	0.0 keV
Fraction of transmitted electrons, $f_{trans}{}^{a}$	0
Average density of the ice mixture, ρ	1.20 g cm^{-3}
Irradiated area, A	1.0 ± 0.2 cm^2
total number of molecules processed	4.5 ± 0.5 × 10^{17}
dose per molecule, D	methylamine: 18.3 ± 0.2 eV
	oxygen: 18.9 ± 2.3 eV
Total number of electrons	2.2 ± 0.2 × 10^{15}

[a] Values from CASINO simulations.

Supplementary Table 2. (a) Infrared features of methylamine-oxygen ice mixture before irradiation and (b) new vibrational bands appeared after irradiation at 5 K.

wavenumber observed (cm^{-1})	wavenumber literature (cm^{-1})[1]	assignments	carrier	Ref.
**(a) Before Irradiation**				
3376	3332	ν_{10}	NH_2 asymm. stretch	(1)
3274	3260	ν_1	NH_2 symm. stretch	(1)
3185	3191		H-bonding	(1)
2950	2942	ν_{11}	CH_3 stretch	(1)
2886	2881	ν_2	CH_3 stretch	(1)
2794	2793	ν_3	CH_3 symm. stretch	(1)
1623	1651	ν_4	NH_2 scissor	(1)
1479	1467	ν_5	CH_3 deformation	(1)
1459	1441	ν_6	CH_3 deformation	(1)
1149	1182	ν_7	CH_3 rocking	(1)
1043	1048	ν_8	C-N stretch	(1)
900	955	ν_9	NH_2 wagging	(1)
**(b) After Irradiation**				
3680-3000	3700-3000	ν (-OH/-NH-)	O-H/N-H stretch	(6)
3000-2800	3000-2800	ν ($-CH_3/-CH_2-$)	C-H stretch	(6)
2343	2343	ν_3 (CO_2)	C=O stretch	(7)
2169	2137	ν_1 (CO)	C≡O stretch	(7)
1693	1662	ν (-C=N-)	C=N stretch	(8)
1612	1616	δ (-NH-)	N-H deformation	(9)
1556		δ (-OH)	O-H bending	
1039		ν (C-N/N-N)	C-N/N-N stretch	

Supplementary Table 3. Parameters for the vacuum ultraviolet (VUV) light generated in the present study

**Photon energy (eV)**		**9.50**	**9.10**
Nd:YAG (ω_1)	Wavelength (nm)	532	355
Dye laser (ω_1)	Wavelength (nm)	606.948	445.132
Dye		Rh 610/ Rh 640	Coumarin 450
ω_2	Wavelength (nm)	449.794	579.014
Nd:YAG (ω_2)	Wavelength (nm)	355	532
Dye laser (ω_2)	Wavelength (nm)	449.794	579.014
Dye		Coumarin 450	Pyrromethane 593
	Nonlinear medium	Kr	Xe

Supplementary Table 4. Calculated adiabatic ionization energies (IE) and relative energies (E_{rel}) of distinct CH_5ON isomers. Values calculated at the CCSD(T)/CBS//CCSD(T)/aug-cc-pVTZ level of theory (blue color text) are considered for comparison with the experiment

		DFT/CCSD(T)		CCSD(T)		
Structure	Name	IE (eV)[a]	E_{rel} (kJ mol^{-1})[b]	IE (eV)[c]	IE range (eV)[d]	E_{rel} (kJ mol^{-1})[e]
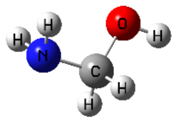	C_s-aminomethanol **(1a)**	9.39	0.00	9.28	9.30 – 9.23	0
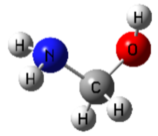	C_1-aminomethanol **(1b)**	9.41	2.28	9.30	9.32 – 9.25	2
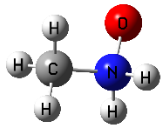	methanamine oxide **(2)**	8.97	243.05	8.82	8.84 – 8.77	239
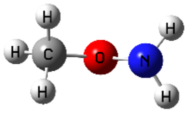	O-methylhydroxylamine **(3)**	8.74	181.28	8.70	8.72 – 8.65	173
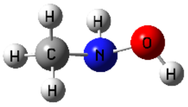	*E*-N-methylhydroxylamine **(4a)**	8.63	156.63	8.55	8.57 – 8.50	151
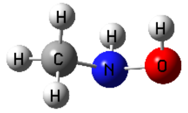	*Z*-N-methylhydroxylamine **(4b)**	8.49	169.79	8.41	8.43 – 8.36	165
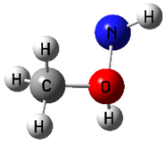	(methyloxonio)amide **(5)**	7.69	434.24	7.58	7.60 – 7.53	420

[a] Adiabatic ionization potential by CCSD(T)/CBS with B3LYP/aug-cc-pVTZ ZPVE correction in eV.
[b] Relative energy by CCSD(T)/CBS with B3LYP/aug-cc-pVTZ ZPVE correction in kJ mol^{-1}.
[c] Adiabatic ionization potential by CCSD(T)/CBS from the CCSD(T)/aug-cc-pVTZ geometry and with ZPVE correction in eV.
[d] IE range is determined by including the error limits (-0.05 to +0.02 eV) from ref. 10 in the calculated (CCSD(T)/CBS//CCSD(T)/aug-cc-pVTZ) IE values and subtracting 0.03 eV to correct for the electric field effect.
[e] Relative energy by CCSD(T)/CBS from the CCSD(T)/aug-cc-pVTZ geometry with ZPVE correction in kJ mol^{-1}.

Supplementary Table 5. Coordinates of the transition states depicted in Supplementary Figure 5

TS (1a →formaldehyde + ammonia)			
	X	Y	Z
C	-0.258986	0.615775	0.000000
O	-1.108789	-0.428744	0.000000
H	0.206646	-1.007593	0.000000
H	-0.244251	1.249930	-0.898680
H	-0.244251	1.249930	0.898680
N	1.063102	-0.211535	0.000000
H	1.631315	-0.140041	0.838061
H	1.631315	-0.140041	-0.838061

TS (1a →methanimine + water)			
	X	Y	Z
C	-0.086560	-0.097928	-0.103223
O	0.025315	0.088776	1.674187
H	1.064594	-0.217021	1.094081
H	0.082160	1.036320	1.877013
H	-0.581014	0.800034	-0.471556
H	-0.724120	-0.971654	-0.044800
N	1.241594	-0.313321	-0.249228
H	1.702148	0.546533	-0.536568

Supplementary References

1. Frigge, R. *et al.* A vacuum ultraviolet photoionization study on the formation of N-methyl formamide ($HCONHCH_3$) in deep space: A potential interstellar molecule with a peptide bond. *Astrophys. J.* **862**, 84, (2018).
2. Jones, B. M., Kaiser, R. I. & Strazzulla, G. UV-VIS, infrared, and mass spectroscopy of electron irradiated frozen oxygen and carbon dioxide mixtures with water. *Astrophys. J.* **781**, 85, (2014).
3. Jones, B. M. & Kaiser, R. I. Application of reflectron time-of-flight mass spectroscopy in the analysis of astrophysically relevant ices exposed to ionization radiation: Methane (CH_4) and D4-methane (CD_4) as a case study. *J. Phys. Chem. Lett.* **4**, 1965-1971, (2013).
4. Bennett, C. J. *et al.* High-sensitivity Raman spectrometer to study pristine and irradiated interstellar ice analogs. *Analytical Chemistry* **85**, 5659-5665, (2013).
5. Davies, M. & Spiers, N. A. in *Adv. Molecular Spectrosc.* (ed A. Mangini) 761 (Pergamon, 1962)
6. Socrates, G. Infrared and Raman characteristic group frequencies. Tables and charts, John Wiley and Sons, Ltd, Chichester, Third Edition, 2001.
7. Zhu, C., Turner, A. M., Abplanalp, M. J. & Kaiser, R. I. Formation and high-order carboxylic Acids (RCOOH) in interstellar analogous ices of carbon dioxide (CO_2) and methane (CH_4). *Astrophys. J. Suppl. Ser.* **234**, 15, (2018).
8. Zhu, C. *et al.* A vacuum ultraviolet photoionization study on the formation of methanimine (CH_2NH) and ethylenediamine ($NH_2CH_2CH_2NH_2$) in low temperature interstellar model ices exposed to ionizing radiation. *Phys. Chem. Chem. Phys.* **21**, 1952-1962, (2019).
9. Zheng, W. & Kaiser, R. I. Formation of hydroxylamine (NH_2OH) in electron-irradiated ammonia−water ices. *J. Phys. Chem. A* **114**, 5251-5255, (2010).
10. Turner, A. M., Chandra, S., Fortenberry, R. C. & Kaiser, R. I. A Photoionization reflectron time-of-flight mass spectrometric study on the detection of ethynamine ($HCCNH_2$) and 2H-Azirine (c-H_2CCHN). *ChemPhysChem* **22**, 985-994, (2021).
