# Supplementary material for: Experimental identification of aminomethanol (NH2CH2OH)—the key intermediate in the Strecker Synthesis
Source: Nat Commun. 2022 Jan 19;13:375. doi: 10.1038/s41467-022-27963-z (PMC8770675; doi:10.1038/s41467-022-27963-z)
Supplement: Supplementary file 3 — Description of Additional Supplementary Files [file 41467_2022_27963_MOESM3_ESM.docx]

**Description of Additional Supplementary Files**

**File name: Supplementary Data 1**

Description: This file includes optimized geometrical coordinates of distinct CH_5_NO isomers calculated at the CCSD(T)/aug-cc-pVTZ level of theory.

**File name: Supplementary Data 2**

Description: This file includes calculated vibrational frequencies (in cm^-1^) of CH_5_NO isomers (**1-5**) at the CCSD(T)/aug-cc-pVTZ level of theory.
