# Supplementary material for: Experimental identification of aminomethanol (NH2CH2OH)—the key intermediate in the Strecker Synthesis
Source: Nat Commun. 2022 Jan 19;13:375. doi: 10.1038/s41467-022-27963-z (PMC8770675; doi:10.1038/s41467-022-27963-z)
Supplement: Supplementary file 4 — Supplementary Data 1 [file 41467_2022_27963_MOESM4_ESM.docx]

**Supplementary Data 1.** Optimized geometrical coordinates of distinct CH_5_ON isomers calculated at the CCSD(T)/aug-cc-pVTZ level of theory.

| *C_s_*-aminomethanol (**1a**) | | | |
| --- | --- | --- | --- |
|  | X | Y | Z |
| H | 0.0000000000 | 0.2216002534 | -1.9248230511 |
| O | 0.0000000000 | -0.3168408413 | -1.1256114710 |
| C | 0.0000000000 | 0.5608590186 | 0.0132290153 |
| H | -0.8917070636 | 1.1949260222 | -0.0067264596 |
| H | 0.8917070636 | 1.1949260222 | -0.0067264596 |
| N | 0.0000000000 | -0.1926719101 | 1.2318484835 |
| H | 0.8162058326 | -0.7940472158 | 1.2648313370 |
| H | -0.8162058326 | -0.7940472158 | 1.2648313370 |

| *C_1_*- aminomethanol (**1b**) | | | |
| --- | --- | --- | --- |
|  | X | Y | Z |
| C | 0.0072309750 | -0.5620630889 | 0.0019689722 |
| O | 0.0293331902 | 0.2330362158 | 1.1716615786 |
| H | -0.6352712980 | 0.9192800324 | 1.0370905786 |
| H | 0.8972908785 | -1.1945130474 | 0.0748057544 |
| H | -0.8796933420 | -1.2057318911 | -0.0264261199 |
| N | -0.0341640886 | 0.2903980650 | -1.1758170858 |
| H | 0.8829125544 | 0.6733427222 | -1.3778231681 |
| H | -0.3422657432 | -0.2291641794 | -1.9897416235 |

| methanamine oxide (**2**) | | | |
| --- | --- | --- | --- |
|  | X | Y | Z |
| H | 0.0000000000 | 0.4154129107 | -2.0797437473 |
| C | 0.0000000000 | -0.2627823892 | -1.2213323349 |
| N | 0.0000000000 | 0.5093911106 | 0.0485186267 |
| H | -0.8892046516 | -0.8886365892 | -1.2093887121 |
| H | 0.8892046516 | -0.8886365892 | -1.2093887121 |
| H | -0.8209038493 | 1.1275511410 | 0.0526788091 |
| H | 0.8209038493 | 1.1275511410 | 0.0526788091 |
| O | 0.0000000000 | -0.3049454072 | 1.1511607997 |

| O-methylhydroxylamine (**3**) | | | |
| --- | --- | --- | --- |
|  | X | Y | Z |
| H | 0.0000000000 | 0.4167992595 | -2.0340484728 |
| C | 0.0000000000 | -0.2665924749 | -1.1834363973 |
| O | 0.0000000000 | 0.5590654024 | -0.0261500449 |
| H | -0.8919501310 | -0.8991476433 | -1.2137141692 |
| H | 0.8919501310 | -0.8991476433 | -1.2137141692 |
| N | 0.0000000000 | -0.3107738803 | 1.1269306940 |
| H | 0.8106164218 | 0.0013457306 | 1.6593053069 |
| H | -0.8106164218 | 0.0013457306 | 1.6593053069 |

| *E*-N-methylhydroxylamine (**4a**) | | | |
| --- | --- | --- | --- |
|  | X | Y | Z |
| C | 0.0002816212 | 0.2746061204 | -1.2112632343 |
| N | -0.0789070148 | -0.5867310683 | -0.0312136192 |
| O | 0.0643788582 | 0.2838004925 | 1.1202779010 |
| H | -0.7228404106 | 0.0746622140 | 1.6335194799 |
| H | 0.7740333884 | -1.1404604064 | 0.0051923540 |
| H | 0.0666918289 | -0.3733892571 | -2.0889209028 |
| H | -0.9114300006 | 0.8685054815 | -1.2794465043 |
| H | 0.8648006542 | 0.9469289091 | -1.1852914903 |

| Z-N-methylhydroxylamine (**4b**) | | | |
| --- | --- | --- | --- |
|  | X | Y | Z |
| C | -0.0013297503 | 0.2730321485 | -1.2062964754 |
| N | -0.0573732084 | -0.5856928703 | -0.0282548702 |
| O | -0.0439515678 | 0.2356308423 | 1.1489226526 |
| H | 0.7125216995 | 0.8366864822 | 1.0587262249 |
| H | 0.7941893265 | -1.1419459839 | 0.0200363038 |
| H | 0.0680642930 | -0.3676783238 | -2.0884630354 |
| H | -0.9211983613 | 0.8552417592 | -1.2599595740 |
| H | 0.8572070793 | 0.9628863483 | -1.2002772477 |

| (methyloxonio)amide (**5**) | | | |
| --- | --- | --- | --- |
|  | X | Y | Z |
| C | -0.0044363917 | 0.3302814795 | -1.1915856406 |
| O | -0.0541473023 | -0.5684299577 | -0.0562522214 |
| N | 0.0662847576 | 0.3297013826 | 1.2729195931 |
| H | -0.7671694270 | -0.0655137736 | 1.7248655682 |
| H | 0.7826503899 | -1.0463711377 | 0.0070527811 |
| H | 0.1164552847 | -0.2707781702 | -2.0947593962 |
| H | -0.9546070216 | 0.8546002278 | -1.1851553511 |
| H | 0.8139193945 | 1.0335483577 | -1.0486521561 |
