# Supplementary material for: Experimental identification of aminomethanol (NH2CH2OH)—the key intermediate in the Strecker Synthesis
Source: Nat Commun. 2022 Jan 19;13:375. doi: 10.1038/s41467-022-27963-z (PMC8770675; doi:10.1038/s41467-022-27963-z)
Supplement: Supplementary file 5 — Supplementary Data 2 [file 41467_2022_27963_MOESM5_ESM.docx]

**Supplementary Data 2**. Calculated vibrational frequencies (in cm^-1^) of CH_5_NO isomers (**1-5**).

| **Normal modes** | **1a** | **1b** | **2** | **3** | **4a** | **4b** | **5** |
| --- | --- | --- | --- | --- | --- | --- | --- |
| ν1 | 221 | 241 | 256 | 146 | 264 | 280 | 213 |
| ν2 | 358 | 356 | 431 | 233 | 314 | 384 | 236 |
| ν3 | 464 | 531 | 881 | 455 | 435 | 450 | 361 |
| ν4 | 864 | 830 | 888 | 875 | 851 | 830 | 574 |
| ν5 | 941 | 964 | 1023 | 1028 | 976 | 957 | 785 |
| ν6 | 1008 | 1011 | 1172 | 1176 | 1060 | 1074 | 1000 |
| ν7 | 1097 | 1121 | 1178 | 1195 | 1153 | 1147 | 1079 |
| ν8 | 1249 | 1217 | 1356 | 1252 | 1228 | 1217 | 1176 |
| ν9 | 1271 | 1312 | 1401 | 1314 | 1366 | 1372 | 1350 |
| ν10 | 1395 | 1389 | 1432 | 1464 | 1443 | 1442 | 1362 |
| ν11 | 1440 | 1455 | 1480 | 1488 | 1483 | 1476 | 1461 |
| ν12 | 1538 | 1543 | 1509 | 1518 | 1486 | 1478 | 1483 |
| ν13 | 1675 | 1652 | 1666 | 1648 | 1523 | 1516 | 1510 |
| ν14 | 3026 | 3013 | 3041 | 3016 | 3015 | 2934 | 3055 |
| ν15 | 3069 | 3057 | 3148 | 3084 | 3093 | 3065 | 3153 |
| ν16 | 3502 | 3503 | 3180 | 3124 | 3125 | 3109 | 3193 |
| ν17 | 3584 | 3595 | 3300 | 3432 | 3469 | 3458 | 3387 |
| ν18 | 3812 | 3799 | 3335 | 3518 | 3820 | 3643 | 3792 |
